# Clinical Application of Circular RNAs as Biomarkers in Acute Ischemic Stroke

**DOI:** 10.3390/jpm13050839

**Published:** 2023-05-16

**Authors:** Chiara Siracusa, Niccolò Vono, Maria Benedetta Morano, Jolanda Sabatino, Isabella Leo, Ceren Eyileten, Eleonora Cianflone, Marek Postula, Daniele Torella, Salvatore De Rosa

**Affiliations:** 1 Department of Medical and Surgical Sciences, Magna Graecia University, 88100 Catanzaro, Italy; chiara.siracusa@unicz.it (C.S.); vono.niccolo@gmail.com (N.V.); cianflone@unicz.it (E.C.); 2 Department of Children and Woman’s Health, University of Padua, 35121 Padua, Italy; jolanda.sabatino@unipd.it; 3 Royal Brompton and Harefield Hospitals, Guy’s and St Thomas’ NHS Foundation Trust, London SW3 5NP, UK; i.leo@rbht.nhs.uk; 4 Department of Experimental and Clinical Medicine, Magna Graecia University, 88100 Catanzaro, Italy; dtorella@unicz.it; 5 Centre for Preclinical Research and Technology, Department of Experimental and Clinical Pharmacology, Medical University of Warsaw, 02-097 Warsaw, Poland; ceren.eyileten-postula@wum.edu.pl (C.E.); mpostula@wum.edu.pl (M.P.); 6 Genomics Core Facility, Center of New Technologies, University of Warsaw, 00-927 Warsaw, Poland

**Keywords:** stroke, ischemia, cerebrovascular disease, noncoding RNAs, circRNAs, biomarkers

## Abstract

Despite the substantial improvement in diagnosis and treatment within the last decades, ischemic stroke still represents a challenge, responsible still for a high burden of morbidity and mortality. Among the unmet clinical needs are the difficulties in identifying those subjects with the greatest risk of developing a stroke, the challenges in obtaining a timely diagnosis, the prompt recognition of the different clinical forms of stroke, the assessment of the response to treatments and the prognostic assessment. All these issues might be improved with appropriate smart biomarkers that could better inform clinical management. The present article offers an overview of the potential role of circular RNAs as disease biomarkers in stroke. A systematic approach was adopted to gather all potentially relevant information in order to provide a panoramic view on this class of promising molecules.

## 1. Introduction

Ischemic stroke (IS) is one of the leading causes of morbidity, disability, and death in the world. IS occurs when cerebral blood flow is interrupted, resulting in loss of brain function. Despite the acute treatment of IS having substantially improved recently, one of the major clinical challenges for available therapies to maintain their efficacy is a timely diagnosis. In this context, there is a strong drive to develop novel tools for the early detection of acute IS (AIS), to assess the efficacy of treatments and for prognostic stratification [1,2,3,4].

Circular RNA (circRNA) is a class of non-coding RNA (ncRNA) characterized by a highly stable and preserved closed loop form. CircRNAs can carry out their biological function by interacting with RNA binding proteins or by modulating the stability of microRNA (miRNA). In addition, circRNAs control gene expression through a variety of mechanisms, including sorting by sponging miRNA, forming ternary complexes with proteins, and coding proteins. CircRNAs play a significant role in regulating genetic expression by influencing the progression of some diseases and modulating specific pathways. The expression and modulation of ncRNAs play a key role in developing and advancing neurological diseases, including IS and brain damage from cerebral ischemia-reperfusion [5,6,7,8,9]. CircRNAs are involved in pathogenic processes such as excitotoxicity, oxidative stress, neuroinflammation, and apoptosis, which may cause secondary brain damage and prevent functional recovery in patients suffering from AIS [10,11].

CircRNA are single-strand molecules of RNA, which are omnipresent in all species, from viruses to mammals. They were originally identified as viruses, pathogens of some plants, in 1976, by Sanger and his colleagues. They are produced through a back-splicing process. Unlike linear RNAs, circRNAs are actually formed by covalent binding of site 5 from an exon upstream to site 3 from the same exon or an exon downstream. To date, two different models of circRNA biogenesis have been described, the lariat or exon skipping model and the direct back-splicing model. In the lariat pattern, canonical splicing occurs prior to splicing back, while in the direct back splicing pattern the circRNA is generated first. Some RNA-binding proteins (RBPs) could act as regulatory activators or inhibitors in circRNA biogenesis by interacting with specific binding sites in the flanking intronic sequences of pre-mRNA [12,13].

**Figure 1:**
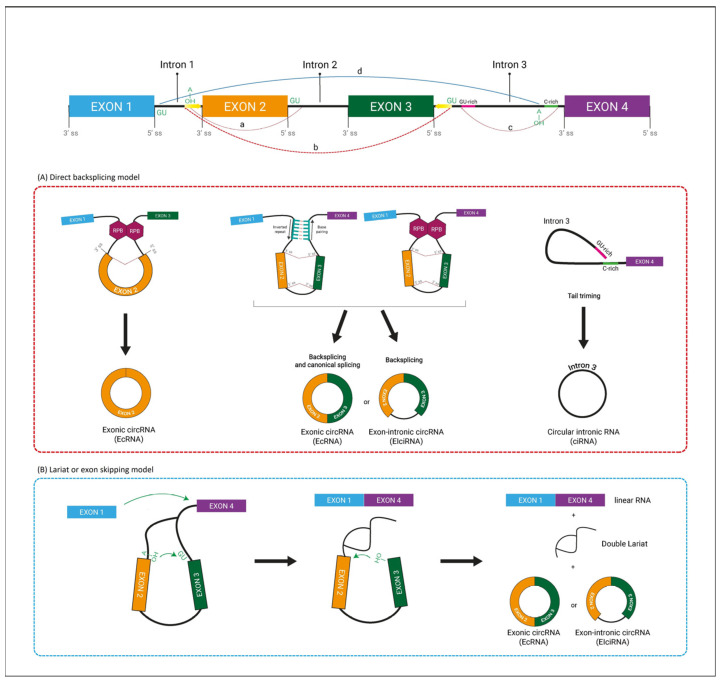
Biogenesis and functions of circRNAs. (**A**) During the development of a pre-mRNA transcript, a lariat structure comprising exons may arise, which is subsequently spliced internally to release introns and generate EcRNAs made of exons. The downstream 3′ splicing donor site in the exon binds to the upstream 5′ splicing acceptor site, resulting in base pairing of the donor and receptor sites, which mediates exon circularization and the formation of circRNAs or more specifically EcRNAs. It can be controlled by ALU or RBPs. Exon-Intron circRNA (EIciRNA) can be generated regardless of whether an intron is present in the splicing mechanism. After the lariat undergoes internal reverse splicing, the interconnections of introns result in the production of circRNAs. (**B**) Circular RNA biogenesis can happen via a lariat precursor, which includes cleaving the precursor at the 5′ splice site (5′ss) and forming a 2′-5′ phosphodiester bond between the guanosine residue at the 5′ end of the intron and an adenosine within the intron.

CircRNAs can be classified into three major groups: exonic circRNAs (EcRNAs), esonintron circRNAs (EIciRNAs), and circular intronic RNAs (ciRNAs). Additional subgroups, intergenic and intronic tRNA circRNAs, are small and rarely studied (Figure 1) [14]. The predominant circRNA is exonic and is found mainly in the cytoplasm. Intronic circRNAs are retained in the nucleus and participate as transcriptional regulators of their respective parental genes by interacting with RNA polymerase II. CircRNAs are found predominantly in the cytoplasm. The lack of a 5′ cap and a 3′ tail makes the circular molecules more resistant to RNase degradation, which is relevant for their stability in extracellular environments. CircRNAs are widely distributed in various organs and tissues, such as the heart, intestine, liver, lung, pancreas, skeletal muscle, skin, adipose, adrenal, and blood vessels [15].

Most circRNAs act as miRNA sponges to regulate endogenous genetic expressions through the rival RNA network. By means of direct interaction with miRNAs, circRNAs play an important role in the regulation of gene expression by influencing the progression of diseases by modulating regulatory pathways. In addition, they can function as protein decoy, thanks to their ability to facilitate the translocation of proteins into the nucleus. CircRNAs can promote parental gene transcription through binding to RNA pol II or U1 snRNP, and then the circRNA with IRES elements or m6A-induced ribosome engagement sites can be translated into proteins (Figure 1).

In this review, we present current evidence and discuss the possible exploitation of circRNAs as disease biomarkers in IS in order to improve diagnosis and prognostic assessment [16,17].

## 2. Systematic Literature Search and Selection of Results

**Figure 2:**
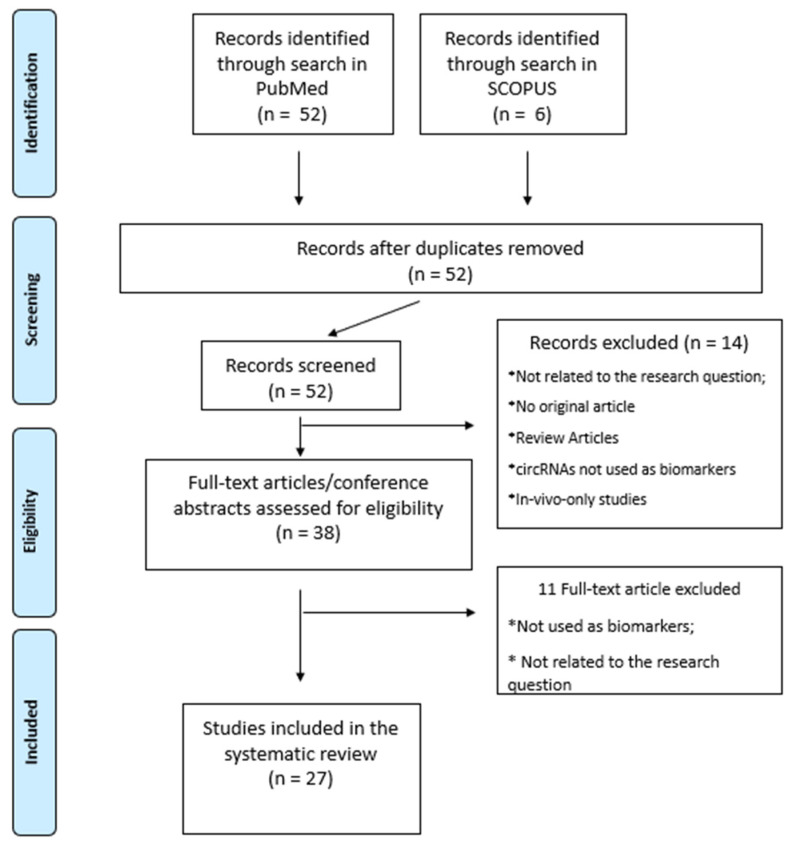
PRISMA flowchart. Article selection flowchart, according to PRISMA guideline. The asterisk (*) refers to all studies excluded from the review because they did not fall into the categories described.

A systematic review was carried out according to the PRISMA (Preferred Reporting Items for Systematic Reviews and Meta-Analysis) guidelines to provide a comprehensive review of available literature on the potential of circRNAs as clinical biomarkers. We conducted a literature search of the PubMed and Scopus databases using the terms “rna, circular” (MeSH Terms) OR (“rna” (All Fields) AND “circular” (All Fields) OR “circular rna” (All Fields) OR “circrna” (All fields) OR “circrnas” (All fields)) AND (“stroke” (MeSH terms) OR “stroke” (All fields) OR “strokes” (All fields) OR “stroke s” (All fields) AND (“biomarker s”(All Fields) OR “biomarkers” (MeSH Terms) OR “biomarkers” (All Fields) OR “biomarker” (All Fields). We included ex-vivo experimental studies to identify the circRNAs most implicated in diagnosis, progression, prognosis, and the therapeutics of stroke. Among search results, articles to be included were selected according to the PRISMA protocol by two researchers independently, as already described [14]. The protocol for this systematic review has been registered on the https://aspredicted.org/repository of Pennsylvania University, accessed on 2 January 2023 (registration number: 120535). The process of selecting articles, from research results to selection, is described in detail in the PRISMA flowchart in Figure 2. Altogether, 27 studies fulfilling the selection criteria were finally included in our systematic review. The studies selected were classified according to the experimental design into specific categories, as reported in Table 1. Specifically, the following categories were used: (i) mechanistical refers to studies which use circRNA to identify the pathophysiological mechanisms of the disease; (ii) explorative refers to studies designed to define an exploratory analysis of the expression profile in a given pathology; (iii) discovery refers to studies designed to identify new molecules to be potentially used as biomarkers; (iv) validation refers to studies designed to validate the association between specific biomarker molecules and the clinical condition in which use should be applied.

## 3. CircRNAs as Diagnostic Biomarkers in Ischemic Stroke

AIS accounts for about 80% of strokes and is characterized by the occlusion of the cerebral artery with disruption of blood flow and consequent loss of neuronal function. In order to ensure better management of the patient with acute cerebrovascular disease, it is necessary to make a prompt and accurate diagnosis, followed by timely treatment, in order to stop the progression of the disease [43,44,45,46,47,48]. Therefore, it is important to develop new strategies to improve the efficacy of available treatment and clinical outcomes, such as (i) risk stratification for the characterization of patients at high risk of AIS; (ii) timely diagnosis, possibly earlier than with currently available strategies; (iii) monitoring of treatment efficacy and guidance of early neuroprotection to optimize the recovery of neural tissue after IS; (iv) individualized treatment selection and patient management; (v) better prediction for treatment response and treatment selection.

**Figure 3:**
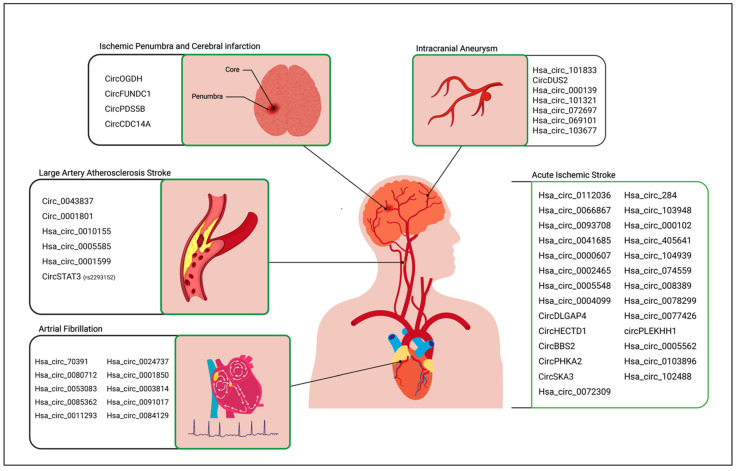
Potential areas of clinical exploitation for circRNAs in stroke.

CircRNAs are expressed within the central nervous system and contribute to the regulation of multiple biological pathways. Thus, they are promising candidates to be used as “smart” clinical biomarkers, providing insights from the basic mechanisms underlying stroke and the pathophysiological pathways activated by the disease and its treatment. In this regard, several studies have already explored the modulation of circRNA expression profiles in body fluids of patients with IS, demonstrating an involvement of these ncRNAs in the pathogenesis of brain damage (Figure 3).

A study performed on plasma samples of patients with AIS found that 96 circRNAs (52 were upregulated and 44 were downregulated) and 683 mRNAs (448 were upregulated and 235 were downregulated) were differentially expressed in patients with IS, compared with healthy controls. In particular, the expression of circSKA3 was significantly higher among IS patients versus healthy controls and positively associated with MMP9 mRNA expression.

Expression of CircOGDH was selectively up-regulated in mouse penumbra tissue with medium cerebral artery occlusion and plasma in 45 patients with AIS, showing a 54-fold increase over non-cerebrovascular disease controls. Interestingly, CircOGDH expression in the brain tissue was closely related to expression in mouse serum with middle cerebral artery occlusion. In particular, CircOGDH was strongly expressed in plasma exosomes in AIS patients as compared to non-vascular patients. These findings demonstrate that CircOGDH is a potential therapeutic target for the regulation of ischemic neural viability and is enriched in exosomes derived from neurons in peripheral blood, acting as a predictive penumbra biomarker in patients with AIS [49]. Exosomes are vesicles of a nanometric size (30–150 nm) consisting of lipid double-layer membranes, present in the blood and secreted by various cell types [18,50]. Exosomes are able to release proteins, lipids, DNA, miRNA and ncRNA in the cellular microenvironment of the receiving cells [51,52], playing a crucial role in cellular communication and cell signal transduction. In recent times, exosomes have been the focus of attention for their potential role as potential targets for the pathogenesis of neurological and cardiovascular diseases and tools for diagnosis and therapy [53]. Xiao et al. [19] identified 25 differentially expressed exo-somal circRNAs in LAA stroke. Of these, nine circRNAs were increased and 16 circRNAs decreased compared to controls. In another study, enrolling both LAA (n = 196) and small artery occlusion (SAO) strokes (n = 170), compared to controls (n = 149), the authors found exo-somal circ_0043837 and circ_ 0001801 had better diagnostic efficacy for LAA stroke than plasma circRNAs. Using these data, the authors built a nomogram showing that integrating circRNAs and known risk factors was able to predict high risk of plaque rupture [20]. A transcriptome-level investigation of the differential expression of circRNAs in patients with LAA stroke revealed hsa_circRNA_0001599 as a potential circRNA biomarker for the diagnosis of LAA stroke, whose expression levels were positively correlated with National Institutes of Health Stroke Scale scores (NIHSS) and heart attack volumes [21].

CircRNAs can act as a sponge to specific miRNAs, such as those involved in regulating the process underlying the development of AIS. These include pathways of activation of RNA and protein, focal adhesion, and trans-endothelial migration of leucocytes, all involved in the pathogenesis of brain damage. It was recently observed that most of the modulated circRNAs bind to EIF4A3 and AGO2, two RNA-binding proteins (RBP) that play a role in the pathophysiology of AIS. In addition, 4 circRNAs (hsa_circ_0112036, hsa_circ_0066867, hsa_circ_93708 and hsa_circ_41685) were found to interact with PI3K-Akt, AMPK, chemokine pathways, and endocytosis, whose perturbation is known in AIS, and which might therefore be potentially helpful as a diagnostic biomarker [54].

Similar findings were reported by Xu et al. [22], who highlighted the alterations of plasma exo-circRNAs in patients with AIS compared with healthy controls by high-performance sequencing. The RNA-seq results showed that a total of 3540 circRNAs, including 1177 circRNAs which increased and 2363 which decreased, had a significant differential expression in patients with AIS compared to the controls. Furthermore, bioinformatic analyses suggest that plasma exo-somal circRNAs may be actively implicated in the mechanism of AIS by regulating biological pathways such as the MAPK signaling pathway, the neurotrophic signaling pathway, the PI3K/Akt pathway, the cGMP-signaling pathway (PKG), the mTor and the p53 signaling pathways.

CircRNAs can also interfere with endocytosis, energy metabolism, apoptosis, platelet activation, neurotrophin signaling and the VEGF signaling pathway, which are indeed involved in the onset of cerebral infarction. In this regard, Xu et al. [11] showed that circRNA are differential expressed in brain infarction, identifying 10 circRNAs which are significantly upward regulated, and 10 circRNAs which are significantly downward regulated. Specifically, hsa_circRNA_000581, hsa_circRNA_092476, hsa_circRNA_101836 and hsa_circRNA_102183 in the acute stroke group were significantly less regulated and hsa_circRNA_103372 was significantly better regulated than the control group. From bioinformatics analysis conducted by Li et al., two circRNAs (hsa_circ_0000607 and hsa_circ_0002465) were seen to be altered and significantly down-regulated in AIS. These results are intriguing, as prediction analyses indicate that hsa_circRNA_0000607 might act as a sponge for miR-337-3p, that in turn targets Bcl2. In fact, the miR-337-3p/Bcl2 axis is known to play a role in AIS [23].

Zheng et al. conducted a case-control study, including 40 patients with AIS within 24 h from the acute onset and 40 healthy subjects as the control group. Results have shown that patients with AIS have lower expression levels of hsa_circ_0004099. The NIHSS score and stroke time were negatively correlated with hsa_circ_0004099 levels, suggesting its potential as therapeutic target in AIS [24].

## 4. CircRNA as Prognostic Biomarkers in Ischemic Stroke

Recent evidence is emerging on the potential use of specific circRNAs for predicting the long-term prognosis and survival of AIS patients or as markers of specific complications during the disease course (Figure 3).

Recent studies provided evidence of single nucleotide polymorphisms (SNPs) associated with circRNA expression. Liu et al. showed that circ-STAT3 rs2293152 is a predictor of functional outcomes after stroke, and the GG genotype showed worse outcomes 3 months after stroke. They identified that genotype GG was associated with an increased risk of adverse outcomes of stroke, while genotype CC + CG was associated with a better outcome at 3 months. Subgroup analysis revealed that the negative effect of genotype GG rs2293152 was more pronounced in women, elderly patients, and people with hypertension. Furthermore, the interaction of age, gender, blood pressure and rs2293152 improved the prediction of poor recovery after AIS. The impact of circ-STAT3 rs2293152 on the short-term prognosis of stroke appears to be mediated by the regulation of circ-STAT3 expression due to its influence on neuroinflammation processes following a neural lesion [25].

CircSKA3 has been shown to be positively associated with poor clinical results; in fact, patients with IS and higher levels of circSKA3 were more likely to have higher triglycerides, higher BMI and a shorter interval from the onset of stroke to hospitalization. Besides its potential utility for diagnosis and as a marker for AIS, circSKA3 unveils an association between blood lipid levels and vascular remodeling, with potential implications for an early and aggressive lipid-lowering approach, as recently claimed for patients with acute coronary syndromes [26,55].

Chen et al. used a novel approach to identifying circRNA-disease associations based on networks of convolutional relationship graphs (the convolutional network method of relationship graphs for circRNA-disease associations, RGCNCDA). The method fully uses circRNA similarity, miRNA similarity, disease similarity, and known association information among three biological entities to construct a global heterogeneous network [27].

Plasma levels of three circRNAs, circFUNDC1, circPDS5B and circCDC14A, were significantly increased in patients with AIS compared to healthy controls, and their levels were positively correlated with the volume of cerebral infarction. An opposite trend was found in AIS patients with favourable outcomes versus poor outcomes, illustrating that the change rate in circRNAs within the first 7 days of treatment could serve as a potential biomarker for predicting stroke outcome [28,29]. Therefore, the immediate detection of circFUNDC1 levels in peripheral blood within 24 h of the onset of patients with AIS can better reflect the severity and outcome of neurological deficits. circFUNDC1 may act as a potential biomarker to detect the elevated risk of stroke-related infection (SAI) in affected patients in order to quickly choose antibiotic treatment. Significantly elevated levels of circFUNDC1 were observed in patients with SAIs compared to patients with non-infectious AIS. Additionally, a positive correlation was found between the circFUNDC1 level and the number of neutrophils. Leukocyte and neutrophil reports were significantly elevated in patients with IS compared to patients without IS, affecting the immune status of patients with stroke and contributing to the development of stroke-related infection [30].

## 5. CircRNAs as Biomarkers in Intracranial Aneurysm

Intracranial aneurysm (IA) is an irregular dilatation of the intracranial artery, usually caused by structural damage of the vessel walls induced or facilitated by inflammation, apoptosis, phenotypic changes of vascular smooth muscle cells, cellular adhesion, atherosclerosis, or the abnormal metabolism of the extracellular matrix. Chen et al. studied circRNA profiles in human-IA tissues compared to the tissues of the superficial temporal artery (STA) and conducted bioinformatic analysis to explore the potential involvement of circRNA in IA. Hundreds of circRNAs with a differential expression pattern in STA compared to other tissues were detected. In particular, the circRNA-101833 was significantly overexpressed [31]. Similarly, a further study with IA patients showed 235 differentially expressed circRNAs. Specifically, 150 circRNAs were up-regulated and 85 down-regulated in IA versus the control group. In addition, seven circRNAs were up-regulated and three were down-regulated in patients with unruptured IA (UIA) versus ruptured IA (RIA). The results suggested that circRNAs were associated with IA formation by modulating the signaling pathway Mtor, again linking circRNAs to vascular remodeling [32].

## 6. CircRNAs as Biomarkers in Atrial Fibrillation and Thromboembolic Stroke

Atrial fibrillation (AF) is a chronic disease, characterized by an alteration of the heart rhythm leading to chaotic atrial activation, which is associated with both an impairment of its mechanical function and an increase in the risk of thrombosis and thromboembolism, the incidence of which has considerably increased in recent decades, with multiple implications for clinical management, particularly because it is frequently associated with other comorbidities [56,57,58]. One of the major challenges is the estimation of the risk of thromboembolic stroke in these patients. Thus, novel, more effective and sensitive biomarkers are strongly sought after. Several studies have focused on the role of circRNAs in the development and onset of atrial fibrillation, in order to identify biomarkers by providing a new approach to AF diagnosis and treatment (Figure 3).

Zhai et al. collected tissue samples from the human right atrial appendix from five patients with persistent AF (AF group) and five patients with normal sinus rhythm (NSR group) and studied differential expression of circRNAs and interactions between circRNA and miRNA. They found about 600 differentiated circRNAs related to AF. In particular, 30 circRNAs (eight up-regulated circRNAs and 22 down-regulated circRNAs) were predicted to possibly act as sponges of nine miRNA [33]. As validation studies are ongoing, the potential application of these findings is currently limited to patients undergoing surgical procedures.

On the other hand, detection of circRNAs from peripheral blood might find faster and easier clinical exploitation. In this regard, a recent study found an upregulation of hsa_circ_70391 in AF patients, which was positively correlated with the degree of left atrial fibrosis (r = 0.88, *p* < 0.001). On the contrary, the expression levels of hsa_circ_0003935 were downregulated. Very intriguing, plasma levels of hsa_circ_70391 were significantly higher in persistent AF compared to paroxysmal AF, suggesting a possible role in AF progression [34].

Given the partial overlap between risk factors for cerebrovascular disease and for thromboembolic risk in AF, identifying the actual etiology of stroke in patients with AF is frequently a clinical dilemma. In this context, a recent study found a differential expression profile for 219 blood-borne circRNAs between patients with atherothrombotic and cardioembolic stroke. In particular, further functional analysis revealed interactions between the deregulated hsa_circRNA_102488 and a number of miRNA involved in stroke-related pathways, such as fatty acid biogenesis or lysine degradation [35].

The potential for circRNAs as a disease biomarker in AF is not limited to the detection of differential etiologies. In fact, some circRNAs under investigation as potential biomarkers for stroke, such as circDLGP4, circHECTD1, circFUNDC1, circPDS5B, and circCDC14A, might reveal useful information about the severity and associated clinical risk. In particular, Zhu et al. [36] studied the expression of circ-DLGAP4 in patients with AIS and evaluated its correlation with the risk and severity of AIS. In patients with AIS, circ-DLGAP4 was down-regulated and miR-143 sponging was able to down-regulate HECTD1 expression, leading to a reduction of pathological damage. Conversely, circHECTD1 expression was increased in the plasma of AIS patients compared with the control group. CircHECTD1 works as an endogenous miR-142 sponge to inhibit miR-142 activity, causing the inhibition of TCDD-inducible poly (ADP-ribose) polymerase (TIPARP) expression with subsequent inhibition of astrocyte activation via macro-autophagy/autophagy [37].

## 7. CircRNA for Differential Diagnosis and Precise Pathophysiological Characterization

Lu et al. explored the expression profile of circRNA in blood in mice subjected to focal cerebral ischemia validation and in AIS patients. The authors showed that 128, 198, and 789 circRNAs were altered at 5 min, 3 h, and 24 h post-stroke, respectively. Specifically, circBBS2 and circPHKA2 were expressed differently in the blood of patients with AIS, demonstrating that blood circRNAs can serve as potential biomarkers for the diagnosis of AIS [38]. Although these data are still at their pre-clinical stage, these results pave the way to a possible exploitation of circRNAs as markers of the age of the ongoing acute cerebral ischemia.

Dong et al. studied the expression of circRNAs in peripheral blood mononuclear cells (PBMCs) of five patients with AIS and five healthy subjects. A total of 521 circRNAs were differentially expressed between AIS patients and healthy controls, 373 circRNAs upregulated and 148 circRNAs downregulated in AIS patients compared to controls. From gene ontology analyses, altered circRNAs were found to target many pathophysiological processes of AIS, particularly with regards to inflammation and immunity. Differentially expressed circRNAs in PBMCs can be helpful as diagnostic biomarkers or potential therapeutic targets [39].

The rupture of the atherosclerotic plaque in the carotid involves a stroke and a myocardial infarction. Bazan et al. measured serum levels of miR-221 and circR-284 in 24 asymptomatic and 17 patients with acute carotid endarterectomy (CEA). MiR-221 was inferior while circR-284 was elevated in the serum of the symptomatic group versus the asymptomatic group. The circR-284:miR221 serum ratio was significantly high in the symptomatic group and had favourable biomarker characteristics, indicating carotid plaque rupture and stroke [40].

Zhai et al. observed that the expression of circ-0072309 was decreased while the content of miR-100 was decreased and increased in serum of AIS patients, demonstrating that there is a negative difference between the expression of circ-0072309 and miR-100. In particular, circ-0072309 could serve as a potential target for the treatment of IS, due to its ability to directly bind miR-100, and to regulate cell survival and apoptosis through Mtor pathway activation [41]. These findings are particularly interesting, as miR-100 has also been related to plaque destabilization in the coronary district.

Silva et al. studied the overall expression of circRNAs in human IS lesion tissue and identified five circRNAs that were differently expressed compared to healthy brain tissue. These circRNA have been shown to be highly accurate and are potential biomarkers of IS. Functional enrichment analysis has suggested their involvement in significant processes in the development of neurodegenerative diseases, including synaptic components and mitochondrial transmission, inflammation and pathways [42]. Interestingly, there is considerable overlap with the circRNAs found to be regulated in blood samples from stroke patients.

## 8. Conclusions

AIS is a leading cause of death and disability worldwide. The study of the molecular mechanisms underlying the development of cerebral ischemia can provide new insights to better inform therapeutic strategies and, more generally, its clinical management, in order to counteract its deleterious clinical impact and improve patients’ prognosis.

Despite recent advances in the therapeutic management of AIS, timely intervention remains a challenge. In this regard, a relevant clinical unmet need it the lack of new and effective biomarkers for clinical implementation.

Multiple studies have demonstrated how modulation of specific circRNAs correlates with several pathophysiological processes underlying IS, including infarct volume, brain oedema, stroke severity, and clinical outcomes. In particular, the alteration of the expression levels of specific circRNAs can be easily measured from plasma samples, which might provide key information, useful for the diagnosis, clinical classification, monitoring, response to treatment and prognosis of AIS. For instance, the expression levels of circFUND1 are positively correlated to the volume of the brain infarct. In particular, in patients with AIS relative to healthy controls, increased circFND1 levels were observed and its detection in the blood within 24 h of the onset of patients with AIS can be predictive of the severity and outcome of neurological deficits. These results broaden our understanding of how PBMC’s circ-DLGAP4 could serve as a potential diagnostic biomarker for AIS. Analysis of the ROC curve has shown that the expression of PBMC circ-DLGAP4 has good predictive value in identifying lower AIS risks. Furthermore, the negative correlation between the expression of PBMC circ-DLGAP4 and several inflammatory markers, as well as the NIHSS score and the CRP (C-reactive protein) level, further indicate the potential of this circRNA as a biomarker of AIS. In addition, the negative correlation between the expression of PBMC circ-DLGAP4 and miR-143 in AIS patients further highlighted its potential as a biomarker for disease surveillance. Overall, these findings suggest that PBMC circ-DLGAP4 expression could be used as a new biomarker to diagnose AIS and for disease surveillance during the post-acute follow up. This exemplifies the usefulness of summoning different sources of evidence to generate new research hypotheses, to be then tested in further research. In this example, this further research could open the way to clinical application.

Along the same lines, CircOGDH might be exploited as a non-invasive biomarker to identify the presence and size of the ischemic penumbra in AIS patients. The rapid identification of the penumbra is crucial to timely transfusion therapy used to save ischemic brain tissue. CircOGDH as a biomarker could enable clinicians to assess the presence and extent of the penumbra quickly and accurately and to facilitate more effective treatment decisions. The high sensitivity and specificity of CircOGDH expression in estimating the penumbra represents a promising option to reduce the need for repeated imaging and to shorten the time to reperfusion therapy. The available evidence constitutes a mature knowledge background for the design of future research to explore the potential clinical application of CircOGDH as a clinical biomarker in AIS patients.

Altogether, experimental results available so far are indeed very promising. However, new pragmatic clinical evidence is expected to define their implementation in clinical practice. Given the acute clinical context, a pragmatic trial focusing on the impact of the adoption of circRNAs as novel biomarkers on time-to-treatment and clinical outcomes in a real-world fashion might be an appropriate option.

## Figures and Tables

**Table 1:** Schematic description of the research articles selected.

Study	Disease Subtype	Type of Study	Detection Methods	Sample	Objectives	Findings
Xiao-Lei Hu, 2021 [11]	IS	Discovery and Validation	RNA sequencing; RNA identification and differential expression analysis; qRT-PCR; computer-based prediction of circRNAs/miRNAs/mRNAs interaction	Blood Samples ACI (n = 3) NC (n = 3)	Study of the regulatory role of circRNAs during ischemic stroke by analyzing the expression profile of those after ischemic stroke and investigate whether they could serve as potential novel biomarkers for early detection of cerebral infarction.	In this study, 10 circRNAs that were up- and down-regulated were chosen and validated by qRT-PCR. The main piece of evidence is that circRNAs are potential biomarkers for cerebral infarction early diagnosis and are up- or down-regulated in the pathogenesis of ACI.
Yanfang Liu, 2022 [16]	AIS	Explorative, Discovery	RNA-sequencing; circRNA Microarray; Differentially Expressed circRNAs; qRT-PCR; Bioinformatics analysis	Blood Samples AIS (n = 45) NC (n = 32)	Investigate whether circOGDH may be a potential biomarker for penumbra in patients with AIS and its role in ischemic neuronal damage.	CircOGDH expression is enriched in neuron-derived exosomes in peripheral blood, making it a potential therapeutic target for controlling ischemia neuronal viability. As a potential biomarker for penumbra detection, circOGDH expression levels in blood showed a positive correlation with the size of cerebral penumbra tissue in the brains of patients with AIS. Additionally, CircOGDH controls the expression of COL4A4 by sponging miR-5112, which is essential for controlling neuronal viability in ischemic conditions.
Shengnan Li, 2021 [18]	LAA-type acute ischemic stroke	Discovery	RNA sequencing; differential expression analysis of circRNAS; qRT-PCR	Blood Sample	Characterize the circRNA expression profile present in LAA-type acute ischemic stroke patients, and to detect suitable biomarkers for LAA-stroke detection.	The NIHSS scores and infarct volumes were positively correlated with the expression levels of Hsa-circRNA-0001599. The study takes hsa-circRNA-0001599 into consideration as a potential circRNA biomarker for diagnosing LAA-stroke.
Qi Xiao, 2021 [19]	LAA stroke	Discovery	RNA sequencing; qRT-PCR; differential expression Analysis of circRNAs, miRNAs and mRNAs;	Blood sample LAA (n = 37) NC (n = 37)	Report a comprehensive expression of circRNAs within exosomes present in peripheral blood during LAA stroke.	The biological roles of their miRNA sponges and encoding proteins in the progression of LAA were discovered by circRNA-ceRNA networks and translatable analysis. The biological and potential diagnostic roles of exo-circRNAs of LAA may be revealed by this research.
Qi Xiao, 2022 [20]	LAA stroke	Mechanistical	RNA sequencing; RT-qPCR	Blood Samples AIS (n = 366) NC (n = 149)	Identification of potential diagnostic and predictive biomarker for LAA stroke and plaque rupture by sequencing and validating exo-somal circRNA.	Exo-somal circRNAs (circ_0043837 and circ_0001801) and circulating exo-somal circRNAs profiling identify novel predictive biomarkers for LAA stroke and plaque rupture with superior diagnostic value to plasma circRNAs. According to this study, circRNAs may serve as biomarkers for plaque rupture.
J. Yang, 2022 [21]	AIS	Mechanistical	circRNA microarray; differential expression analysis of circRNAs; qRT-PCR, circRNA/miRNA/mRNA interaction networks construction	Blood Samples AIS (n = 10) NC (n = 10)	Investigate on circRNA profile of plasma exosomes in AIS patients.	This study shows that circRNAs from plasma exosomes were quantified differently in AIS patients compared to controls. Importantly these results revealed that exo-somal circRNAs from plasma (especially hsa_circ_0112036, hsa_circ_0066867, hsa_circ_0093708, and hsa_circ_0041685) potentially participate in the progression of AIS via sponging miRNAs/RBPs or encoding proteins, may be explored as biomarkers for the diagnosis of AIS or may also be potential targets for therapeutic interventions.
Bingyi Xu, 2022 [22]	IS	Mechanistical	RNA sequencing; analysis of differential expressed circRNAs	Blood Samples IS (n = 3) NC (n = 3)	Study of circRNAs as potential diagnostic biomarkers and therapeutic targets for IS pathogenesis.	According to bioinformatics analysis, signaling pathways linked to IS pathology are enriched in circRNAs and some mRNAs that are differentially expressed. especially the PI3K/Akt, cGMP-PKG, mTOR, p53, and MAPK signaling pathways, as well as the neurotrophic, cGMP-PKG, and PI3K/Akt pathways. The current study shows that the expression profile of the circRNA in plasma exosomes has changed, and it may have an impact on IS. CircRNAs are promising biomarkers for determining damage caused by IS.
Shenghua Li, 2020 [23]	AIS	Explorative, discovery	RNA-seq; Bioinformatic analysis: CIRI2, Find_circ software; qRT-PCR	Blood Sample AIS (n = 3) NC (n = 3)	Investigate circRNA expression profiles in AIS in the South Chinese Han population.	hsa_circ_0005548 was upregulated and hsa_circ_0000607 and hsa_circ_002465 was downregulated in AIS patients, according to three randomly chosen circRNAs (among 2270). Hsa_circ_0000607 may be essential for the development and pathogenesis of AIS by controlling the miR-337-3p/Bcl2 axis and the apoptosis pathway.
J. Zheng, 2022 [24]	AIS	Mechanistical	RT; Qpcr; miRNA-circRNA interaction analysis; miRNA target genes analysis	Blood Samples (plasma) AIS (n = 40) NC (n = 40) circRNA gene assay from GEO database	Investigate the expression and effect of hsa_circ_0004099 in AIS and provide a possible molecular target for AIS treatment.	The current study showed that hsa_circ_0004099 expression is low in AIS patients. The expression levels of hsa_circ_0004099 were negatively correlated with the NIHSS score and infarct time, indicating that it might be a potential therapeutic target for enhancing AIS outcomes. The AUC of hsa_circ_0004099 is 92.3%, demonstrating its potential use in the diagnosis and prognosis of AIS and indicating a good predictive value for AIS.
Xu Liu, 2021 [25]	IS	Mechanistical	Database research, PCR-LDR (polymerase chain reaction and ligation detection reaction); bioinformatics analysis	No clinical samples	Study the association between circRNAs polymorphism related to inflammation and functional outcome.	The circ-STAT3 polymorphism (rs2293152) may be a novel biomarker for assessing functional outcomes following stroke and a key factor in the recovery from ischemic stroke.
Tian Xu, 2021 [26]	AIS	Explorative	RNA sequencing; RNA identification and differential expression analysis; qRT-PCR	Blood sample IS (n = 220) NC (n = 62)	Screen and evaluate the prognostic value of circRNAs that influence MMP9 through the competing endogenous RNA (ceRNA) network.	This investigation demonstrated how circSKA3 worked as a ceRNA for hsa-miR-6796-5p to accelerate the development of IS by targeting MMP9. Additionally, poor IS results were positively correlated with baseline circSKA3. When considered as a whole, the data may demonstrate that circSKA3 is a potential biomarker or therapeutic target in IS.
Y. Chen, 2022 [27]	General Diseases	Mechanistical	Machine Learning Models; Relational Graph Convolutional Network	No samples were analyzed CircRNA-disease association data, miRNA-disease association data and circRNA-miRNA interaction data	Propose a new computational method to predict circRNA-disease associations based on relational graph convolutional networks (R-GCNs).	In order to create a global heterogeneous network, the RGCNCDA (relational graph convolutional networks circRNA-disease associations) method fully exploits the similarities between three biological entities—circRNA, miRNA, disease, and known associations between them.
Lei Zuo, 2020 [28]	AIS	Discovery and Validation	circRNA microarray; qRT-PCR; differential expression analysis of circRNAs	Blood Sample AIS (n = 36) NC (n = 36)	Identify and validate differentially expressed circRNAs in stroke patients and investigate their potential as biomarkers for the diagnosis and prognosis of AIS.	In this study, 3 up-regulated circRNAs were identified. The expression levels of circFUNDC1, circPDS5B, and circCDC14A were all positively correlated with infarct volume. These three circRNAs combined may be a biomarker for identifying and forecasting the course of strokes.
J. Zu, 2022 [29]	AIS	Mechanistical	qRT-PCR; Qpcr with specific primers for different circRNAs	Blood Samples AIS (n = 200) NC (n = 100)	Explore the potential value of circRNAs for identifying acute neurological deterioration and estimating long-term survival for AIS.	This study showed that the etiological types of strokes (LAA, SAO, and CE) differed in their elevated circFUNDC1 levels, and that circFUNDC1 levels were higher in patients with END (early neurological deterioration). Additionally, it has been shown that the severity and prognosis of neurological dysfunction may be more accurately predicted by the immediate detection of circFUNDC1 levels in peripheral blood within 24 h of the onset of stroke. Additionally, patients’ survival times were shortened when their circFUNDC1 levels were higher. CircFUNDC1 may be able to predict the outcome of the acute phase and the long-term survival of AIS.
Lei Zuo, 2020 [30]	Stroke Associated Infection (SAI)	Discovery	circRNA microarray; differential expression analysis of circRNAs; qRT-PCR	Plasma Sample SAI (n = 26) NON-SAI (n = 42)	Figure out whether circFUNDC1, circPDS5B and circCDC14A could be diagnosis and prognosis biomarkers of SAI.	The levels of circFUNDC1 and WBC and neutrophil counts are positively correlated. It would be advantageous for AIS patients to receive preventive care if CircFUNDC1 were used to build a risk model for the prediction of SAI. CircFUNDC1 has previously been mentioned as a possible AIS biomarker.
Xin Chen, 2021 [31]	IA	Discovery and Validation	circRNA Microarray; differential expression analysis of circRNAs; qRT-PCR,	Intracranial Aneurysm tissue (n = 15) and Superficial Temporary Artery tissue (n = 15, non-IA)	Explore circRNAs underlying IA rupture and search for molecular targets for the prediction and diagnosis of IA rupture.	Because circDUS2 was found to be upregulated in IA tissue compared to STA tissue, this study demonstrates that it is a potential molecular marker for intracranial aneurysm. It also demonstrates that circDUS2 is located in the cytoplasm of brain vascular smooth muscle cells. Furthermore, through its effect on the SMAD family, involvement in the TGF-b signaling pathway, and participation in the MAPK signaling pathway, the upregulation of hsa_circRNA_101833 was linked to the development of IAs.
Yonggang Ma, 2021 [32]	IA	Mechanistical, Discovery	circRNA Microarray; bioinformatics analysis; qRT-PCR	Blood Samples IA (n = 10) NC (n = 5)	Examine how circRNAs regulate the formation of IA and identify the potential molecules contributing to IA rupture.	CircRNAs influence the mTOR signaling pathway, which is linked to the development of IA. Our findings suggested that the mTOR pathway or differentially expressed circRNAs may be latent therapeutic targets for IA, as well as a list of potential diagnostic biomarkers for the disease.
Zhenyu Zhai, 2020 [33]	AF	Mechanistical, discovery	RNA sequencing; Bioinformatic analysis: CIRI2, CIRCexplorer2;	Human Right Atrial Appendage Tissues AF (n = 5) NSR (n = 5) MicroRNA dataset (GSE68475)	Identification of AF-related circRNAs and construction of the integrative regulatory network of circRNAs, microRNAs and mRNAs.	The hub regulatory network in persistent AF was built through interactions between hsa-miR-339-5p and its related circRNAs and target mRNAs, which may shed new light on the potential mechanism underlying persistent AF. These significant molecular components may also develop into new AF diagnostic and therapeutic biomarkers.
F. Wei, 2022 [34]	AF	Discovery	circRNA microarray; qRT-PCR; differential expression analysis of circRNAs	Microarray Expression Profiles from GEO (GSE129409, GSE97455, GSE28954, and GSE115574) Blood Samples AF (n = 80) NC (n = 40)	Construct a circRNA-miRNA-mRNA-mediated network and explore its potential function in AF.	This study discovered a circRNA-miRNA-mRNA network that was strongly linked to AF. A new molecular target for AF has also been identified, and it has been shown that hsa_circ_0070391 was related to left atrial fibrosis and prognosis with AF with radiofrequency catheter ablation.
Aiora Ostolaza, 2020 [35]	IS (different etiology)	Discovery and validation	circRNA microarray; RT-qPCR; functional in silico analysis	Blood Sample (n = 50)	Identify differentially expressed circRNAs in AIS patients according to stroke etiology.	When compared to cardioembolic strokes, a group of 60 circRNAs were found to be upregulated. The expression profiles of circRNAs vary depending on the subtype of stroke. Hsa_circ_102488 RBPs sites grouped around the AGO2 and FUS proteins. A set of miRNAs involved in pathways related to stroke, such as fatty acid biogenesis or lysine degradation, were found to interact with deregulated circRNAs.
Xiaoqi Zhu, 2019 [36]	AIS	Explorative	qRT-PCR; differential expression analysis of circRNAs	Blood Sample AIS (n = 170) NC (n = 170)	Investigate the expression of circ-DLGAP4 and its correlation with severity, inflammation, inflammation cytokine levels and miR-143 expression in AIS patients.	In contrast to controls, PBMC circ-DLGAP4 was down-regulated in AIS patients. Additionally, TNF-a, IL-6, IL-8, and IL-22 serum expression were all negatively correlated with the same. Expression of circ-DLGAP4 in PBMCs was inversely correlated with miR-143 in PBMCs. This data supports the use of circ-DLGAP4 as a new AIS biomarker.
Bing Han, 2018 [37]	Cerebral Ischemic Stroke	Mechanistical	circRNA microarray; qRT-PCR; Bioinformatic Analysis	Blood sample AIS (n = 37) NC (n = 34)	Investigate whether the circHECTD1-MIR142-TIPARP (TCDD induced poly[ADP-ribose]polymerase) axis is involved in cerebral ischemic injury and astrocyte activation.	The study demonstrates that miR-142′s target, TIPARP, was upregulated along with circHECTD1, which served as a sponge for miR-142. Astrocyte activation was caused by the interaction between circHECTD1 and miR-142, which aids in cerebral infarction. According to this study, circHECTD1 will likely serve as a new biomarker and therapeutic target for stroke.
Dan Lu, 2020 [38]	AIS	Discovery	circRNA microarray; differential expression of circRNAs; qRT-PCR	Blood Sample AIS (n = 8) NC (n = 8)	Determine the potential of circRNAs in blood as diagnostic biomarkers for acute ischemic stroke.	Different time points after IS resulted in a significant change in an increasing number of circRNA. The extracellular matrix receptor interaction, fatty acid metabolism, and the Hippo signaling pathway were all connected to the circRNA-targeted genes. Numerous of these circRNAs had human conservation. In the blood of AIS patients, circBBS2 and circPHKA2 were differentially expressed.
Zhaofei Dong, 2020 [39]	AIS	Explorative	RNA sequencing; qRT-PCR; Bioinformatic analysis: CIRI2, CIRCexplorer2; sequence conservation analysis	Blood sample AIS (n = 5) NC (n = 5): Peripheral Blood Mononuclear Cells (PBMCs)	Investigate circRNAs’ expression profiles and function prediction in PBMCs of patients with AIS.	For the first time, the study shows that circRNAs are differentially expressed in the PBMCs of AIS patients and may play a role in the pathogenesis of the disease. These findings suggest possible biomarkers for the detection of AIS and the possibility of investigating therapeutic approaches that specifically target the aberrantly expressed circRNAs.
Hernan A Bazan, 2017 [40]	Plaque Rupture, Ischemic Stroke	Discovery, Validation	qRT-PCR; Droplet Digital PCR	Blood Sample (n = 112 of which asymptomatic (n = 47), acutely symptomatic (n = 41) and symptomatic (n = 24)).	Determine whether in the serum levels of miR-221, miR-222 and circR-284 are observed in patients with asymptomatic high-grade carotid disease versus patients with acutely symptomatic carotid disease and recent IS.	The research demonstrates how recent information may support the development of serum circ-284:miR-221 as a diagnostic biomarker for carotid-related ischemic stroke. Serum circR-284:miR-221 has the potential to function as a diagnostic biomarker for atherosclerotic plaque rupture.
Y. Zhao, 2020 [41]	IS	Discovery	qRT-PCR	Blood sample of serum IS (n = 70) NC (n = 90)	Investigate the expression of circ_0072309 in patients with IS and explore the underlying mechanism of circ_0072309 in IS.	circ_0072309-miR-100-mTOR regulatory axis could alleviate IS
Pablo W Silva, 2020 [42]	IS	Mechanistical	Differential Expression Analysis; functional in-silico analysis	Public RNA-Seq data of Human Ischemic Stroke Lesion Tissues and Controls (GSE56267)	Describe the global expression of circRNAs in human ischemic stroke lesion tissue and provide novel insights of using them as potential biomarkers.	CircRNAs play a role in ataxia, inflammation, and synaptic transmission. Furthermore, the molecular context of stroke may feature prominent roles for hsa_circ_0078299 and FXN. This study suggests that circRNAs are potential therapeutic targets for brain recovery and neuroprotection.

Abbreviations: NC: normal control; AF: atrial fibrillation; AIS: acute ischemic stroke; IA: intracranial aneurysm; IS: ischemic stroke; LAA: large artery athero-sclerotis; GEO: Gene Expression Omnibus (https://www.ncbi.nlm.nih.gov/geo/, accessed on 6 April 2023); SAO: small artery occlusion; CE: cardio-embolism; AUC: area under the curve; RBP: RNA binding protein; CIRI2: CIrcularRNA Identifier (https://sourceforge.net/projects/ciri/, accessed on 6 April 2023); CIRCexplorer2: circular RNA analysis toolset (https://github.com/YangLab/CIRCexplorer2, accessed on 6 April 2023); Find_circ_software: algorithm used for circRNA detection from RNA-sequencing reads.

## Data Availability

No new data were created or analyzed in this study. Data sharing is not applicable to this article.
